# Chemical Composition, Antioxidant and Antihyperglycemic Activities of the Wild *Lactarius deliciosus* from China

**DOI:** 10.3390/molecules24071357

**Published:** 2019-04-06

**Authors:** Zhou Xu, Liang Fu, Shiling Feng, Ming Yuan, Yan Huang, Jinqiu Liao, Lijun Zhou, Hongyu Yang, Chunbang Ding

**Affiliations:** 1 College of Life Sciences, Sichuan Agricultural University, Yaan 625014, China; xzhsicau@163.com (Z.X.); fengshilin@outlook.com (S.F.); yuanming@sicau.edu.cn (M.Y.); shirley11hy@163.com (Y.H.); liaojinqiu630@sicau.edu.cn (J.L.); zhouzhou124@126.com (L.Z.); yhy4868135@163.com (H.Y.); 2 Dazhou Institute of Agricultural Sciences, Dazhou 635000, China; fuliangrain@126.com

**Keywords:** *Lactarius deliciosus*, chemical composition, antioxidant, antihyperglycemic

## Abstract

The wild mushroom *Lactarius deliciosus* from China was studied for the first time to obtain information about its chemical composition, antioxidant, and antihyperglycemic activities. Nutritional value, dietary fiber, fatty acids, metal elements, free sugars, free amino acids, organic acids, flavor 5′-nucleotides, and volatile aroma compounds were determined. Potential antioxidant and antihyperglycemic activities were also tested by investigating 1,1-diphenyl-2-picrylhydrazyl (DPPH) and 2,2′-Azino-bis(3-ethylbenzothiazoline-6-sulfonic acid) (ABTS) radicals scavenging activities, ferric ion reducing activity, as well as α-amylase and α-glucosidase inhibitory activities using ethanol and aqueous extracts. The results showed that *L. deliciosus* was a good wild mushroom with high protein, carbohydrate, and dietary fiber contents, while low in fat and calorie, extensive unsaturated fatty acids contents, with negligible health risks about harmful metal elements. Twenty kinds of free amino acids were detected with a total content 3389.45 mg per 100 g dw. Flavor 5′-nucleotides including 5′-CMP, 5′-UMP, 5′-IMP, and 5′-AMP were 929.85, 45.21, 311.75, and 14.49 mg per 100 g dw, respectively. Mannitol (7825.00 mg per 100 g dw) was the main free sugar, and quininic acid (729.84 mg per 100 g dw) was the main organic acid. Twenty-five kinds of volatile aroma compounds were identified, acids (84.23%) were the most abundant compounds based on content, while aldehydes (15 of 25) were the most abundant compounds based on variety. In addition, both ethanol and aqueous extracts from *L. deliciosus* exhibited excellent antioxidant activity. While in antihyperglycemic activity tests, only ethanol extracts showed inhibitory effects on α-amylase and α-glucosidase.

## 1. Introduction

Edible mushrooms consist of Basidiomycota and Ascomycota members [1]. Abundant protein, essential amino acids, mineral elements, dietary fiber, flavor 5′-nucleotides, and volatile aroma components endowed mushrooms with great nutritional value and unique flavor [2,3]. Thus, mushrooms have been consumed as popular food stuff and flavoring for centuries, and consumer demand has continued to increase in recent years. Nowadays, dozens of cultivated mushrooms such as *Agaricus bisporus*, *Lentinula edodes*, *Pleurotus ostreatus*, *Flammulina velutipes*, and *Auricularia auricula* occupy the main consumer market, but there are far more mushrooms that cannot be artificially cultivated called wild mushrooms that only come from field acquisition [4,5]. Although difficult to collect, wild mushrooms are still pursued by innumerable gluttons due to their unique flavors, and *Tuber melanosporum*, *Tricholoma matsutake* and *Collybia albuminosa*, etc., are regarded as gems [6,7]. Moreover, in the east, traditional medicine theory holds the concept that “drug homologous food”, hence, mushrooms also serve as indispensable raw materials for pharmaceuticals [8].

*Lactarius* Persoon is an ectomycorrhizal group belonging to the family of Russulaceae and widespread in temperate, subtropical and tropical forests. To date, ~500 species of *Lactarius* Per. have been discovered around the world and *Lactarius deliciosus* is one of them [9]. Owing to its excellent taste, texture, and flavor, *L. deliciosus* is popular in China, with some chemical substances and bioactivities of *L. deliciosus* being reported. Research has shown that sesquiterpenoids contribute to the unique color of *L. deliciosus*, some of which possess potent biological activities such as antimicrobial and anticancer [10,11,12]. Ding et al. and Hou et al. reported that *L. deliciosus* polysaccharides exhibited significant anti-tumor activity in mice in vivo and immunomodulatory effects through proliferative growth of both B cell and macrophages cell in vitro [13,14]. Meanwhile, other *L. deliciosus* extracts also have various biological activities such as enzymes inhibition, antioxidant, antimicrobial, and anti-inflammatory [15,16,17].

As an essential class of food raw material, the nutritional value of mushrooms is comprehensively decided by proteins, carbohydrates, fats, minerals, etc., and this is crucial consumer focus point [18]. Other factors such as free sugars, free amino acids, organic acids, flavor 5’-nucleotides, and volatile aroma components could dramatically affect taste and flavor, and should not be ignored [19,20]. In China, although *L. deliciosus* is a popular wild edible mushroom, comprehensive studies of this fungi are relatively lacking. Based on the above, we collected wild *L. deliciosus* from Sichuan Province (South-west of China), and chemical compositions including proteins, carbohydrates, fats, dietary fiber, minerals, free sugars, free amino acids, organic acids, flavor 5′-nucleotides, and volatile aroma components were analyzed. In addition, we evaluated bioactivities by assaying antioxidant and anti-hyperglycemic activities of ethanol and aqueous extracts. 

## 2. Results and Discussion

### 2.1. General Nutritional Value

Generally, mushrooms are considered as a valuable health food since they have perfect proportions of protein, fat, and carbohydrate. As summarized in Table 1, fresh *L. deliciosus* was preponderantly moist, and dry matter content was relatively low (8.00%). This result was in accordance with previous studies demonstrating that dry weight content of fresh mushrooms was generally 5–15% [21,22]. In the dried fruiting body, carbohydrate was the most abundant substance with 66.61 g per 100 g dw, followed by protein (17.19 g per 100 g dw), ash (8.62 g per 100 g dw), and fat (4.82 per 100 g dw). Moreover, 31.81 g per 100 g dw total dietary fiber was detected in *L. deliciosus*, indicating that consuming this mushroom is a great way for dietary fiber intake. On the whole, *L. deliciosus* is a good food that can meet the low-calorie requirements. 

Four fatty acids were identified in the crude fat and their constituents were as follows: palmitic acid (C16:0, 5.17%), stearic acid (C18:0, 16.96%), oleic acid (C18:1, 48.37%), and linoleic acid (C18:2, 29.49%) (Table 1 and Figure S1). Unsaturated fatty acids (C18:1 and C18:2) were dominant in *L. deliciosus* fat, which was in agreement with previous research on wild edible mushrooms [23,24]. Moreover, recent research has documented that unsaturated fatty acids may lower the risk of cardiovascular disease, type two diabetes, and cancer [25,26]. Nevertheless, considering the low level of fat in *L. deliciosus*, the health effects of various fatty acids are very limited.

Compared to green plants, the metal content in mushrooms is higher due to their effective mechanism of easily accumulating metals from the ecosystem [27]. Thus, wild edible mushrooms are regarded as an excellent choice for dietary mineral requirement. Concurrently, a hidden danger is arising with excess heavy metal ingestion. As summarized in Table 2, magnesium and calcium contents of *L. deliciosus* were 1244.29 and 247.07 mg per kg dw, this result was in agreement with a previous conclusion that calcium content in mushrooms is 100–500 mg/kg dw, and magnesium is 800–1800 mg per kg dw, based on data collected from over 1000 samples of 400 mushroom species [28]. Among trace elements, iron content (197.01 mg per kg dw) was notably the highest. Of note, compared with numerous studies, copper content (1.28 mg per kg dw) in this sample was relatively low, which might be related to the physiological property of this species due to *L. deliciosus* always possessing lower copper content in other comparative studies [29,30]. Usually, daily intake is 300 g of fresh mushroom, which contains ~30 g of dry matter [31]. Compared with recommended dietary allowance (RDA) and adequate intake (AI) for females and males (aged from 19 to 30) recommended by the Institute of Medicine, consumption of *L. deliciosus* does not provide significant contribution to calcium and copper supplementation, while good contribution of magnesium, zinc, manganese, iron, and chromium [32,33]. Notably, although a large daily intake value of chromium was observed (up to 344.57% for male and 482.40% for female of AI%), this poses no risk to the human body, with reference to the tolerable upper intake level recommended by the Institute of Medicine [33]. Moreover, toxic arsenic, cadmium, and plumbum were also detected in *L. deliciosus*. EU scientific committee standards stipulated provisional tolerable daily intake values for arsenic, cadmium, and plumbum for adults (of 60 kg body weight) were 0.13, 0.06, and 0.21 mg, respectively. Therefore, the intake of heavy metals (arsenic, cadmium, and plumbum) via consuming *L. deliciosus* is risk-free for the consumers.

### 2.2. Non-Volatile Compounds Relating to Special Flavour

Edible mushrooms are considered a valuable food for their abundance of nutrients and desirable complex delicious taste. The taste of mushrooms is primarily ascribed to abundant soluble non-volatile taste components, such as free amino acids, flavor 5′-nucleotides, free sugars, and organic acids [34,35]. As shown in Table 3 and Figure S2, twenty-two kinds of amino acids have been measured, of which, twenty kinds were found in the free amino acids of *L. deliciosus*, and the content of total free amino acids (TAA) was 3389.45 mg per 100 g dw. Among these amino acids, glutamic acid, glutamine, histidine, and alanine were found in relatively high concentrations. Moreover, the content of nine kinds of essential amino acids (EAA) in *L. deliciosus* was 1026.29 mg per 100 g dw, accounting for 30.28% of total free amino acids. This proportion was approximate to a previous report by Sun et al., in which they found the ratios of EAA/TAA in *Tricholomopsis lividipileata, Boletinus pinetorus*, and *Amanita hemibapha* were 26.82, 27.94, and 34.75%, respectively [36]. Free amino acids can be classified into four groups based on their taste characteristics. Aspartic acid and glutamic acid were monosodium glutamate-like (MSG-like) components responsible for umami taste. The level of MSG-like free amino acids in *L. deliciosus* was 415.71 mg per 100 g dw, which was at low-level (< 500 mg per 100 g dw) of MSG-like components according to the standard defined by Yang et al. [37]. Threonine, serine, glycine, and alanine were sweet taste amino acids, amounting to 734.82 mg per 100 g dw. Valine, methionine, isoleucine, leucine, phenylalanine, histidine, arginine, tryptophan, and tyrosine were classified as bitter amino acids. In *L. deliciosus*, the amount of bitter amino acids was 1033.29 mg per 100 g dw. Although bitter amino acids might bring bitterness, while sweet taste amino acids and soluble sugars could mask this unpleasant taste. Asparagine, glutamine, citrulline, proline, and lysine did not have effects on taste. 

The chromatograms concerning flavor 5’-nucleotides, free sugars, and organic acids are presented in Figures S3–S5, and the compositions of these components are summarized in Table 4. The amount of total flavor 5′-nucleotides in *L. deliciosus* was 1301.30 mg per 100 g dw, and the individual contents of 5′-CMP, 5′-UMP, 5′-IMP, and 5′-AMP were 929.85, 45.21, 311.75, and 14.49 mg per 100 g dw, respectively, while 5′-GMP and 5′-XMP were not detected. Based on the amount of total flavor 5′-nucleotides, Yang et al. divided flavor 5′-nucleotides into three ranges, low (<100 mg per 100 g dw), medium (100–500 mg per 100 g dw), and high (>500 mg per 100 g dw) [37]. Accordingly, the amount of flavor 5′-nucleotides in *L. deliciosus* fell within the high category. Regarding free sugars, trehalose and mannitol have been detected, and their contents were 4990.09 and 7825.00 mg per 100 g dw, respectively. Compared with other wild mushrooms, the content of total free sugars in *L. deliciosus* was in the mid range, which would contribute a moderate sweet taste perception [38,39,40]. In the context of organic acids, quininic acid predominated in *L. deliciosus*, followed by l-malic acid and fumaric acid. Oxalic acid was not found in *L. deliciosus* in this study, in which was however identified in another report of *L. deliciosus* from Portugal [41].

Food flavor is a comprehensive concept that contains sweet, sour, bitter, spicy, astringent, umami, etc. Umami is especially important for edible mushrooms because they are usually used as natural freshness-enhancing materials. Equivalent umami concentration (EUC) value calculated using an equation from sensory evaluation, based on the report of Yamaguchi et al., has often been used to evaluate umami-like taste characteristics of mushrooms [42]. For *L. deliciosus*, the EUC value was 145.32 g per 100 g dw. Mau (2005) grouped mushrooms EUC values into four levels: the first level, >1000 g per 100 g dw, the second level, 100–1000 g per 100 g dw; the third level, 10–100 g per 100 g dw; and the fourth level, <10 g per 100 g dw [43]. Thus, the EUC value for *L. deliciosus* belonged to the second level. 

### 2.3. Volatile Aroma Compounds Relating to Special Flavor

Although mushroom tastes depend on water-soluble non-volatile compounds, the function of volatile aroma compounds could not be ignored due to the fact that they directly influence consumer acceptability. Typical volatile aroma compounds in mushrooms originated mostly from chemical or enzymatic oxidation of unsaturated fatty acids and further interactions with proteins, peptides, and free amino acids [44]. In this research, volatile aroma compounds of *L. deliciosus* were estimated by headspace solid phase micro-extraction GS-MS combining library catalog. As shown in Table 5 and Figure S6, 25 compounds were identified, including 15 aldehydes, six acids, two alkanes, one alcohol, and one ester. Based on qualitative and quantitative analysis, acids were confirmed to be the most important aroma volatile compounds accounting for a 84.23% total chromatographic area, followed by aldehydes with 14.77%. Acids dominated in *L. deliciosus* aroma volatile compounds, which was in accordance with a previous study on *L. edodes* and *Pleurotus sajor-caju* conducted by Çağlarırmak [45]. However, other studies have shown that alcohols are important aromatic substances in mushrooms, which differed to our findings in the present research, this might be caused by the drying process, since Tian also found that drying leads to a sharp drop in alcohols while acids and aldehydes increase in *L. edodes* [44]. Moreover, volatile aroma compounds of mushrooms were also affected by growth conditions and genetic differences.

### 2.4. Antioxidant Activity

Free radicals induce cell-damage, which can cause DNA mutation, proteins damage, lipid peroxidation, and low-density lipoproteins modification. Free radicals can also cause several diseases including diabetes, cancer, neurodegenerative and cardiovascular diseases [46,47]. Usually, food or food extracts can act as antioxidant to counteract damage caused by free radicals. However, the antioxidant capacity of food is determined by complicated factors with various action mechanisms [48]. Thus, several evaluation methods are always used simultaneously to evaluate the antioxidant capacity of food. Due to this effect, we evaluated the antioxidant activity of *L. deliciosus* through DPPH and ABTS radical scavenging and ferric ion reducing (FRAP) assays using ethanol and aqueous extracts. The antioxidant capacity was expressed as trolox equivalent antioxidant capacity (TEAC) values. As shown in Table 6, the TEAC values of ethanol extracts with DPPH, ABT, S and FRAP assays were 18.38, 20.07, and 10.72 μmol Trolox/g dw, respectively. For aqueous extracts, these values were 45.63, 48.05, and 22.28 μmol Trolox/g dw, respectively. Aqueous extracts showed 2–3 fold higher antioxidant capacity compared to ethanol extracts, which might be related to the content of phenols in two extracts, since the total phenol content in aqueous extract was 3.01 fold higher than in ethanol extract. 

### 2.5. Antihyperglycemic Activity

**Figure 1:**
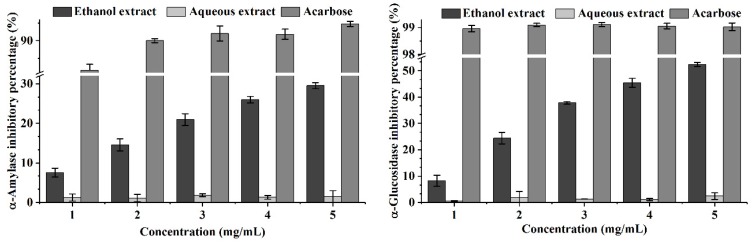
Antihyperglycemic activity of ethanol extract and aqueous extract obtained from wild *L. deliciosus*.

α-Amylase and α-glucosidase are key enzymes in the digestive system that catalyze carbohydrate hydrolysis to enhance blood glucose concentration. A proportion of the population, especially diabetics, suffer from hyperglycemia. A therapeutic approach to hyperglycaemia is to retard the absorption of glucose by inhibiting carbohydrate-hydrolyzing enzymes [49]. Thus, effective and nontoxic inhibitors of α-amylase and α-glucosidase are crucial for the treatment of hyperglycemia. As shown in Figure 1, ethanol extracts exhibited a dose-dependent increase in both α-amylase and α-glucosidase inhibitory assays, while compared with acarbose, the antienzyme activity of ethanol extract was weaker. At 5.0 mg/mL, ethanol extracts exhibited 29.53 and 52.36% on α-amylase and α-glucosidase inhibition, respectively. However, regarding aqueous extracts, no inhibitory effects on α-amylase and α-glucosidase were observed. 

## 3. Materials and Methods

### 3.1. Mushroom Species

Wild growing *Lactarius deliciosus* fruiting bodies were collected from Dazhou (Southwest China) pine forests, in the autumn of 2016. All the samples were selected and authenticated at the Dazhou Institute of Agricultural Sciences based on their microscopic and macroscopic characteristics.

### 3.2. Standards and Reagents

1,1-diphenyl-2-picrylhydrazyl (DPPH), 2,2′-Azino-bis(3-ethylbenzothiazoline-6-sulfonic acid) diammonium salt (ABTS) were purchased from Sigma Chemical Co. (St. Louis, MO, USA). Ferric-tripyridyltriazine (Fe^3+^-TPTZ) is purchased from Beyotime Biotechnology (Shanghai, China). Chromatographic grade analytical standards asparaginic acid, glutamic acid, asparagine, serine, glutamine, histidine, glycine, threonine, citrulline, arginine, alanine, tyrosine, cystine, valine, methionine, tryptophan, phenylalanine, isoleucine, leucine, lysine, hydroxyproline, proline, 5′-CMP, 5′-UMP, 5′-GMP, 5′-IMP, 5′-XMP, 5′-AMP, trehalose, mannitol, quininic acid, l-malic acid and fumaric acid purchased from Solarbio Science & Technology Co., Ltd. (Beijing, China). α-Amylase, α-glucosidase, 4-nitrophenyl α-*d*-glucopyranoside (PNPG), and soluble starch were purchased from Shanghai Yuanye Bio-Technology Co., Ltd. (Shanghai, China). All other reagents were analytical grade and obtained from Chengdu Kelong Chemical Factory (Chengdu, China). 

### 3.3. Nutritional Value Assay

Moisture, ash, crude fat, crude proteins, and dietary fiber of the research sample were analyzed by the Association of Official Analytical Chemists methods [50]. Briefly, moisture content was measured by hot air heating at 105 °C until constant weight; ash content was measured by calcination at 600 ± 15 °C using a muffle furnace; crude fat was measured by Soxhlet extraction method with petroleum; crude protein content was estimated by the macro-Kjeldahl method using convert coefficient as 4.38; dietary fiber content was estimated by enzymatic hydrolysis method. Total carbohydrate content was measured by the phenol-sulfuric acid method after test sample completely hydrolyzed by hydrochloric acid. Total energy contribution was calculated according to the following equation [40]:

(1) Energy (kcal)=4×(g proteins+g carbohydrate)+9×(g fat)

### 3.4. Fatty Acids Composition Assay

Fatty acids composition of crude fat was measured by GC-MS method. Briefly, 20 mg crude fat was methylated with 1 mL of sodium hydroxide (1 M) methanol solution. Then, 2 mL *n*-hexane was added to the mixture. Finally, the *n*-hexane phase was filtered through a 0.22-μm membrane filter before GC-MS analysis. The chromatographic analysis was performed on an Agilent 7890B-5977A GC-MS system equipped with an HP-5MS column (30 m × 0.25 mm × 0.25 μm). Helium was used as carrier gas at the flow rate of 1 mL/min. The injection volume was 1.0 μL with a split ratio of 1:50 at 250 °C. The column temperature was programmed as follow: initial temperature at 50 °C (held for 1 min), increased to 160 °C at 20 °C/min (held for 1 min), increased to 200 °C at 20 °C/min (held for 1 min), increased to 250 °C at 5 °C/min (held for 5 min). Mass spectrometry conditions: interface temperature 250 °C, ion source temperature 230 °C, MS quadrupoles temperature 150 °C, electron energy 70 eV, and m/z scanned area 35–550.

### 3.5. Metal Elements Assay

Metal elements of *L. deliciosus* were analyzed by ICP-MS method. Briefly, 0.2 g lyophilized sample powder was put into digestion tank, 5 mL HNO_3_ was added and fully digested in a Multiwave Pro microwave digester (Anton Paar GmbH, Graz, Austria). Then, excess acid was expelled under 150 °C, and residual liquid was diluted with deionized water to 25 mL. Finally, metal elements analysis was performed on a PerkinElmer NexION350D system (PerkinElmer Co., Waltham, MA, USA). The working parameters were as following: Radio-frequency power 1500 W, nebulizer flow rate 0.8 L/min, coolant gas flow 15 L/min, scanning mode peak hopping, sampling depth 10, and isotopes of selected ^24^Mg^+^, ^43^Ca^+^, ^53^Cr^+^, ^55^Mn^+^, ^57^Fe^+^, ^63^Cu^+^, ^66^Zn^+^, ^75^As^+^, ^111^Cd^+^, ^208^Pb^+^.

### 3.6. Free Sugars and Free Amino Acids Assay

Free sugars and free amino acids were analyzed according to previous methods [23,36] with slight modification. Briefly, 0.2 g lyophilized sample powder was suspended in 20 mL aqueous ethanol (80%, *v*/*v*), and extracted 30 min at 80 °C. The clear supernatant was obtained through centrifuging at 10,000× *g* for 10 min. The above extraction process repeated again, and supernatant was collected together. Then, the supernatant was analyzed by an Agilent 1260 HPLC system equipped with a Hi-Plex Ca column (300 × 7.7 mm, 8.0 µm) and a Refractive Index Detector (RID) for free sugars assay. The analysis conditions of free sugars were as follows: injection volume: 5 μL; mobile phase: H_2_O; flow rate of mobile phase: 0.5 mL/min; column temperature: 80 °C; detector temperature: 40 °C. Furthermore, free amino acids were analyzed by HPLC equipped with a Zorbax Eclipse AAA column (150 × 4.6 mm, 5.0 µm) and a Fluorescence Detector (FLD) (Agilent Technologies, Inc., Santa Clara, CA, USA) using above supernatant by online pre-column derivatization high performance liquid chromatography that has been detailed reported by Sun et al. [36]. 

### 3.7. Flavor 5′-Nucleotides Assay

Flavor 5′-nucleotides were analyzed according to the previous method [51] with slight modification. Briefly, 0.5 g lyophilized sample powder was mixed with 20 mL distilled water and extracted by boiling water bath for 1 min. Then, the clear supernatant was obtained through centrifuging at 10,000× *g* for 10 min. The above extraction process repeated again, and the supernatant was collected together. Finally, flavor 5′-nucleotides were analyzed by HPLC equipped with a Zorbax SB-C18 column (150 × 4.6 mm, 5.0 µm) and a Diode Array Detector (DAD) (Agilent Technologies, Inc., Santa Clara, CA, USA) using above supernatant. The analysis conditions were as follows: injection volume: 10 μL; mobile phase A: H_2_O; mobile phase B: 0.01 M KH_2_PO_4_ (pH = 4.0; containing 1.45 M tetrabutylammonium hydrogen sulfate); mobile phase C: Acetonitrile; mobile phase gradient: 80-80-77-77% of mobile phase B and 3-3-6-6% of mobile phase C along with analysis time linear increased from 0-9.5-13-15 min; flow rate of mobile phase: 1.0 mL/min; column temperature: 25 °C; detector wavelength: 254 nm.

### 3.8. Organic Acids Assay

Organic acids were analyzed by SPE-HPLC method. Briefly, 0.15 g lyophilized sample powder was extracted twice by 20 mL methanol at 40 °C for 30 min. The supernatant was collected through centrifugation and dried under negative pressure condition. Then, the residue was re-dissolved by 1 mL distilled water and purified by SAX solid phase extraction column. Finally, the purified liquid was diluted to 100 mL and analyzed by HPLC equipped with a Zorbax SB-C18 column (150 × 4.6 mm, 5.0 µm) and a Diode Array Detector. The analysis conditions were as follows: injection volume: 10 μL; mobile phase A: 0.1% phosphoric acid solution; mobile phase B: methanol; mobile phase ratio: mobile phase A: mobile phase A = 97.5:2.5; flow rate of mobile phase: 1.0 mL/min; column temperature: 40 °C; detector wavelength: 210 nm.

### 3.9. Equivalent Umami Concentration

The equivalent umami concentration (EUC, g monosodium glutamate (MSG) per 100 g) was used to reflect the umami intensity of *L. deliciosus*, and is represented by the following addition equation according to Yamaguchi report [42].

(2) $$Y=\sum a_{i}b_{i}+1218\left(\sum a_{i}b_{i}\right)\left(\sum a_{j}b_{j}\right)$$

where *Y* is the EUC of the mixture in terms of g MSG/100 g; *a_i_* is the concentration (g per 100 g dw) of each umami amino acid (aspartic acid or glutamic acid); *a_j_* is the concentration (g per 100 g dw) of each umami 5′-nucleotide (5′-IMP, 5′-GMP, 5′-XMP or 5′-AMP); *b_i_* is the relative umami concentration for each umami amino acid to MSG (aspartic acid, 0.077; glutamic acid, 1); *b_j_* is the relative umami concentration for umami 5′-nucelotide to 5′-IMP (5′-IMP, 1; 5′-GMP, 2.3; 5′-XMP, 0.61; 5′-AMP, 0.18); and 1218 is a synergistic constant based on the concentration g per 100 g used.

### 3.10. Volatile Aroma Components Assay

Volatile aroma components were analyzed using the headspace SPME-GC-MS method. Briefly, 2.0 g lyophilized sample powder was put into a sample bottle, a polydimethylsiloxane SPME fiber (100 μm, Supelco, Bellefonte PA) was used to adsorb statically for 40 min at 50 °C. Then, the volatile aroma components were released at 250 °C and analyzed on an Agilent 7890B-5977C GC-MS system equipped with DB-5MS column (30 m × 0.25 mm × 0.25 μm). Helium was used as carrier gas at the flow rate of 1 mL/min. The column temperature was programmed as follow: initial temperature at 40 °C (held for 1 min), increased to 70 °C at 10 °C/min (held for 2 min), increased to 105 °C at 3 °C/min (held for 1 min), increased to 180 °C at 5 °C/min (held for 1 min), increased to 220 °C at 10 °C/min (held for 5 min). Mass spectrometry conditions: interface temperature 280 °C, ion source temperature 230 °C, MS quadrupoles temperature 150 °C, electron energy 70 eV, and *m*/*z* scanned area 35–550.

### 3.11. Ethanol and Aqueous Extracts Preparation

The lyophilized sample was extracted with ethanol at 50 °C for 1 h three times, the supernatant was collected, concentrated and dried to obtain ethanol extracts. Then, the residue was extracted with distilled water at 80 °C for 1 h three times to obtain aqueous extract.

### 3.12. Antioxidant Activity Assay

#### 3.12.1. DPPH Radical Scavenging Activity Assay

DPPH radical scavenging activity was measured according to the previous method described in [52] with slight modification. Briefly, 70 μL sample solution was mixed with 140 μL DPPH-ethanol solution. Then, the mixture was incubated in the dark for 30 min at room temperature, and the absorbance at 517 nm was measured using a spectrophotometric micro-plate reader. Trolox solution was used as a positive control and the antioxidant properties of samples were expressed as μmol trolox per g dry weight extract (μmol Trolox/g dw).

#### 3.12.2. ABTS Radical Scavenging Activity Assay

ABTS radical scavenging activity was measured according to previous method described in [53] with slight modification. Briefly, 100 μL sample solution was mixed with 100 μL ABTS solution. Then, the mixture was incubated in the dark for 6 min at room temperature, and the absorbance at 734 nm was measured. Trolox solution was used as a positive control and the antioxidant properties of samples were expressed as μmol trolox per g dry weight extract (μmol Trolox/g dw).

#### 3.12.3. Ferric Ion Reducing Activity Assay

Ferric ion reducing activity was measured according to previous method described in [54] with slight modification. Briefly, 100 μL sample solution was mixed with 100 μL Ferric-tripyridyltriazine (Fe^3+^-TPTZ) solution. Then, the mixture was incubated in the dark for 10 min at room temperature, and the absorbance at 593 nm was measured. Trolox solution was used as a positive control and the antioxidant properties of samples were expressed as μmol trolox per g dry weight extract (μmol Trolox/g dw).

#### 3.12.4. Total Polyphenols Content Assay

Total polyphenols contents of ethanol and aqueous extracts were determined by the Folin-Ciocalteu assay [55] with some modifications. Briefly, 1 mL sample solution was mixed with 2 mL Folin-Ciocalteu reagent. After incubated for two minutes, 2 mL Na_2_CO_3_ (10%, *w*/*v*) was added and the resulting mixture was incubated for 15 min at 50 °C. Finally, absorbance at 775 nm was measured and polyphenol contents was expressed as mg gallic acid equivalents per g dry weight extract (mg GAE/g dw).

### 3.13. Antihypertensive Activity Assay

#### 3.13.1. α-Amylase Inhibition Activity Assay

α-Amylase inhibition activity was carried out according to previous method [56] with slight modification. Briefly, α-amylase (1 U/mL), soluble starch (1%) and a series concentration of sample were dissolved by 0.02 M sodium phosphate buffer (pH = 6.9, containing 0.0067 M sodium chloride). Sample solution (100 μL) and 100 μL α-amylase solution were mixed and incubated at 37 °C for 10 min. After adding 500 μL soluble starch solution, the mixture was incubated at 37 °C for 10 min. Then, the reaction was terminated by 100 μL hydrochloric acid solution (5 M). After diluting above mixture six times with distilled water, 50 μL liquid was taken, added into 96-well plate, and 150 μL of iodine solution was added as color developing reagent. The absorbance at 660 nm was measured using a spectrophotometric micro-plate reader. Finally, the α-amylase inhibition activity was calculated as the following formula:

(3) $$\alpha -Amylase\;inhibition\;activity\;\left(\%\right)=\frac{Abs_{1}}{Abs_{2}}\times 100$$

where *Abs*_1_ was the absorbance of sample solution mixed with α-amylase solution and soluble starch solution; and *Abs*_2_ was the absorbance of sample solution mixed with soluble starch solution (α-amylase solution was replaced by sodium phosphate buffer).

#### 3.13.2. α-Glucosidase Inhibition Activity Assay

α-Glucosidase inhibition activity was carried out according to previous method described in [57] with slight modification. Briefly, α-glucosidase (0.5 U/mL), 4-nitrophenyl α-d-glucopyranoside (PNPG, 3 mM) a series concentration of sample were dissolved by 0.1 M sodium phosphate buffer (pH = 6.8). Sample solution (100 μL) and 100 μL α-glucosidase solution were mixed and incubated at 37 °C for 10 min. After adding 100 μL PNPG solution, the mixture was incubated at 37 °C for 10 min. Then, the reaction was terminated by 300 μL sodium carbonate solution (0.2 M). Finally, the absorbance at 405 nm was measured using a spectrophotometric micro-plate reader, and the α-glucosidase inhibition activity was calculated as the following formula:

(4) $$\alpha -Glu\cos idase\;inhibition\;activity\;\left(\%\right)=\left[1-\frac{\left(Abs_{1}-Abs_{2}\right)}{Abs_{0}}\right]\times 100$$

where *Abs*_0_ was the absorbance of the α-glucosidase solution mixed with PNPG solution; *Abs*_1_ was the absorbance of sample solution mixed with α-glucosidase solution and PNPG solution; and *Abs*_2_ was the absorbance of sample solution mixed with PNPG solution (α-glucosidase solution was replaced by sodium phosphate buffer).

### 3.14. Statistical Analysis

All assays were carried out in triplicate, and the results are expressed as mean ± standard deviation (SD). 

## 4. Conclusions

In conclusion, chemical analysis, GC-MS, ICP-MS, HPLC, SPE-HPLC, HS-SPME-GC-MS, radicals scavenging assays, ferric ion reducing activity assay, and enzymes inhibitory assays were conducted to evaluate nutritional value, non-volatile flavor compounds, volatile aroma compounds, potential antioxidant and anti-hyperglycemic activities of wild mushroom *L. deliciosus* from China for the first time. Experimental data indicated that *L. deliciosus* is a good wild edible mushroom with high nutritional value, low calorie level, and extensive flavor compounds. Moreover, *L. deliciosus* also have potential as natural antioxidant and anti-hyperglycemic agents in food and pharmaceutical industry in the future. 

## Figures and Tables

**Table 1:** Proximate composition, energetic value, dietary fiber, and fat composition of wild *L. deliciosus*.

Component	*L. deliciosus*
Moisture (g per 100 g)	92.00 ± 0.64
Dry matter (g per 100 g)	8.00 ± 0.64
Total carbohydrate (g per 100 g dw)	66.61 ± 1.02
Crude fat (g per 100 g dw)	4.82 ± 0.15
Crude Protein (g per 100 g dw)	17.19 ± 0.06
Ash (g per 100 g dw)	8.62 ± 0.25
Energy (kcal per 100 g dw)	378.60 ± 2.74
Total dietary fiber (g per 100 g dw)	31.81 ± 1.51
Insoluble dietary fiber (g per 100 g dw)	26.51 ± 1.54
Soluble dietary fiber (g per 100 g dw)	5.30 ± 0.36
C16:0 (% of total fatty acids)	5.17 ± 0.30
C18:0 (% of total fatty acids)	16.96 ± 0.19
C18:1 (% of total fatty acids)	48.37 ± 0.62
C18:2 (% of total fatty acids)	29.49 ± 0.55

**Table 2:** Contents and daily intake estimations of metal elements of wild *L. deliciosus*.

Element	Content (mg per kg dw)	Daily Intake (mg/day) ^a^	RDA or AI (mg/d) ^d^		RDA or AI % ^d^	
			Male	Female	Male	Female
Magnesium	1244.29 ± 42.16	37.33	400 ^b^	310 ^b^	9.33	12.04
Calcium	247.07 ± 4.23	7.41	1000 ^c^	1000 ^c^	0.74	0.74
Zinc	52.34 ± 2.68	1.57	11 ^b^	8 ^b^	14.28	19.63
Manganese	23.12 ± 0.75	0.69	2.3 ^c^	1.8 ^c^	30.16	38.53
Iron	197.01 ± 13.14	5.91	8 ^b^	18 ^b^	73.88	32.84
Chromium	4.02 ± 0.69	0.12	0.035 ^c^	0.025 ^c^	344.57	482.40
Copper	1.28 ± 0.02	0.04	0.9 ^b^	0.9 ^b^	4.27	4.27
Arsenic	0.75 ± 0.04	0.02	-	-	-	-
Cadmium	1.91 ± 0.05	0.06	-	-	-	-
Plumbum	0.85 ± 0.04	0.03	-	-	-	-

^a^ Daily element intake values calculated using 30 g *L. deliciosus* dry matter; ^b^ RDA recommended by the Institute of Medicine; ^c^ AI recommended by the Institute of Medicine; ^d^ RDA or AI for both males and females aged 19–30; - Toxic elements without RDA and AI.

**Table 3:** Free amino acids composition of wild *L. deliciosus*.

Free Amino Acid	Content (mg per 100 g dw)
Aspartic acid ^b^	39.43 ± 0.87
Glutamic acid ^b^	376.29 ± 4.12
Asparagine	79.41 ± 0.84
Serine ^c^	136.35 ± 0.66
Glutamine	794.06 ± 54.85
Histidine ^a,d^	278.57 ± 23.62
Glycine ^c^	91.32 ± 0.59
Threonine ^a,c^	131.76 ± 3.45
Citrulline	16.26 ± 0.59
Arginine ^d^	155.70 ± 11.34
Alanine ^c^	375.38 ± 2.59
Tyrosine ^d^	91.53 ± 1.38
Cystine	-
Valine ^a,d^	122.17 ± 4.93
Methionine ^a, d^	25.41 ± 0.62
Tryptophan ^a, d^	68.95 ± 0.43
Phenylalanine ^a, d^	133.61 ± 0.45
Isoleucine ^a, d^	47.82 ± 0.95
Leucine ^a, d^	109.53 ± 1.61
Lysine ^a^	208.46 ± 16.92
Hydroxyproline	-
Proline	107.43 ± 12.44
Total free amino acids (TAA)	3389.45 ± 38.13

^a^ Essential amino acids; ^b^ Monosodium glutamate-like (MSG-like) amino acids; ^c^ sweet amino acids; ^d^ bitter amino acids; - Not detected.

**Table 4:** Flavor 5′-nucleotides, free sugars and organic acids composition of wild *L. deliciosus*.

Component	Content (mg per 100 g dw)
5′-CMP	929.85 ± 42.33
5′-UMP	45.21 ± 6.72
5′-IMP	311.75 ± 13.43
5′-AMP	14.49 ± 3.37
Trehalose	4990.09 ± 307.95
Mannitol	7825.00 ± 466.72
Quininic acid	729.84 ± 71.80
l-Malic acid	415.63 ± 87.44
Fumaric acid	120.71 ± 11.45

**Table 5:** Aroma volatile compounds of wild *L. deliciosus*.

Component	Composition (%)
Heptanal	2.03 ± 0.15
Benzaldehyde	2.23 ± 0.09
Hexanoic acid	0.67 ± 0.02
Octanal	1.19 ± 0.07
Benzyl alcohol	0.08 ± 0.00
2-Octenal	0.58 ± 0.11
Nonanal	1.58 ± 0.16
2-Nonenal	0.83 ± 0.05
Dodecane	0.29 ± 0.04
Decanal	0.79 ± 0.05
2-Decenal	1.62 ± 0.29
Undecanal	0.46 ± 0.02
2,4-Decadienal	0.29 ± 0.01
2-Undecenal	1.48 ± 0.18
2-Butyl-2-octenal	0.16 ± 0.02
*n*-Decanoic acid	0.76 ± 0.05
Decanoic acid, ethyl ester	0.24 ± 0.01
Tetradecane	0.38 ± 0.06
Dodecanal	0.47 ± 0.08
2-Dodecenal	0.65 ± 0.03
Tridecanal	0.41 ± 0.10
*n*-Hexadecanoic acid	12.82 ± 0.67
9,12-Octadecadienoic acid	3.11 ± 0.21
9-Octadecenoic acid	60.57 ± 2.89
Octadecanoic acid	6.30 ± 0.74

**Table 6:** Antioxidant capacity and total phenols in ethanol and aqueous extracts obtained from wild *L. deliciosus*.

	TEAC _DPPH_ (μmolTrolox/g dw) ^a^	TEAC _ABTS_ (μmolTrolox/g dw) ^b^	TEAC _FRAP_ (μmolTrolox/g dw) ^c^	Total Phenols Content (mg GAE/g dw) ^d^
Ethanol extract	18.38 ± 1.31	20.07 ± 1.75	10.72 ± 1.04	4.55 ± 0.24
Aqueous extract	45.63 ± 4.40	48.05 ± 3.37	22.28 ± 3.25	13.68 ± 0.26

^a^ Trolox equivalent antioxidant capacity (TEAC), 1,1-diphenyl-2-picrylhydrazyl (DPPH); ^b^ 2,2′-Azino-bis(3-ethylbenzothiazoline-6-sulfonic acid) diammonium salt (ABTS); ^c^ Ferric ion reducing antioxidant power (FRAP); ^d^ Gallic acid equivalent weight (GAE).

## Supplementary Materials

The following are available online, Figure S1: Gas chromatography mass spectrum of fatty acids of *L. deliciosus* fat; Figure S2: High performance liquid chromatography of amino acids analyzed by online OPA-FMOC derivation; Figure S3: High performance liquid chromatography of flavor 5’-nucleotides; Figure S4: High performance liquid chromatography of organic acids; Figure S5: High performance liquid chromatography of free sugars; and Figure S6: Headspace solid phase micro-extraction gas chromatography mass spectrum of aroma volatile compounds from *L. deliciosus*.
