# Supplementary material for: Chemical Composition, Antioxidant and Antihyperglycemic Activities of the Wild Lactarius deliciosus from China
Source: Molecules. 2019 Apr 6;24(7):1357. doi: 10.3390/molecules24071357 (PMC6479662; doi:10.3390/molecules24071357)
Supplement: Supplementary file 1 [file molecules-24-01357-s001.pdf]

molecules

MDPI

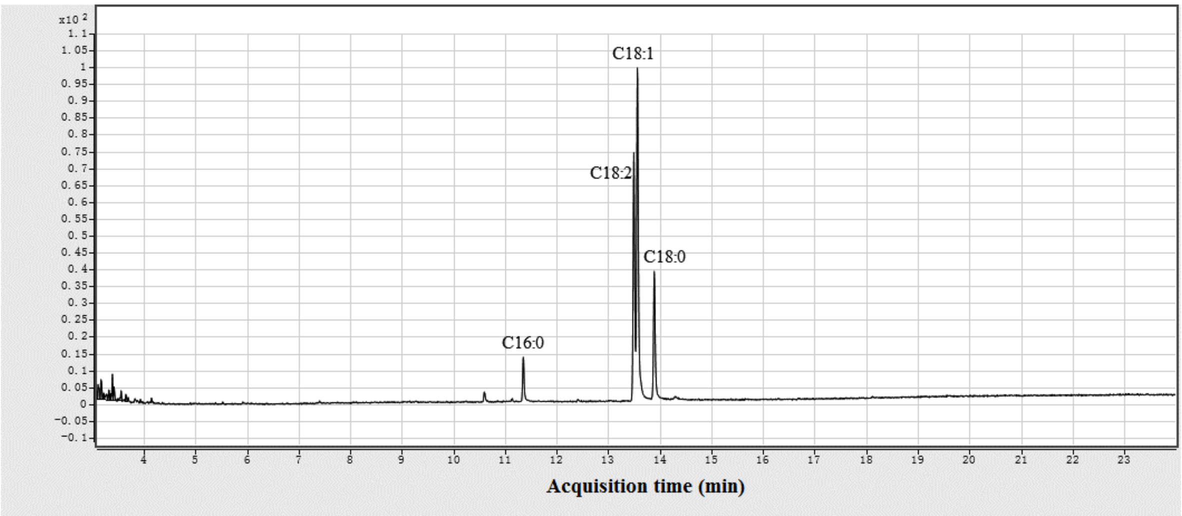

Figure S1. Gas chromatography mass spectrum of fatty acids of *L. deliciosus* fat.

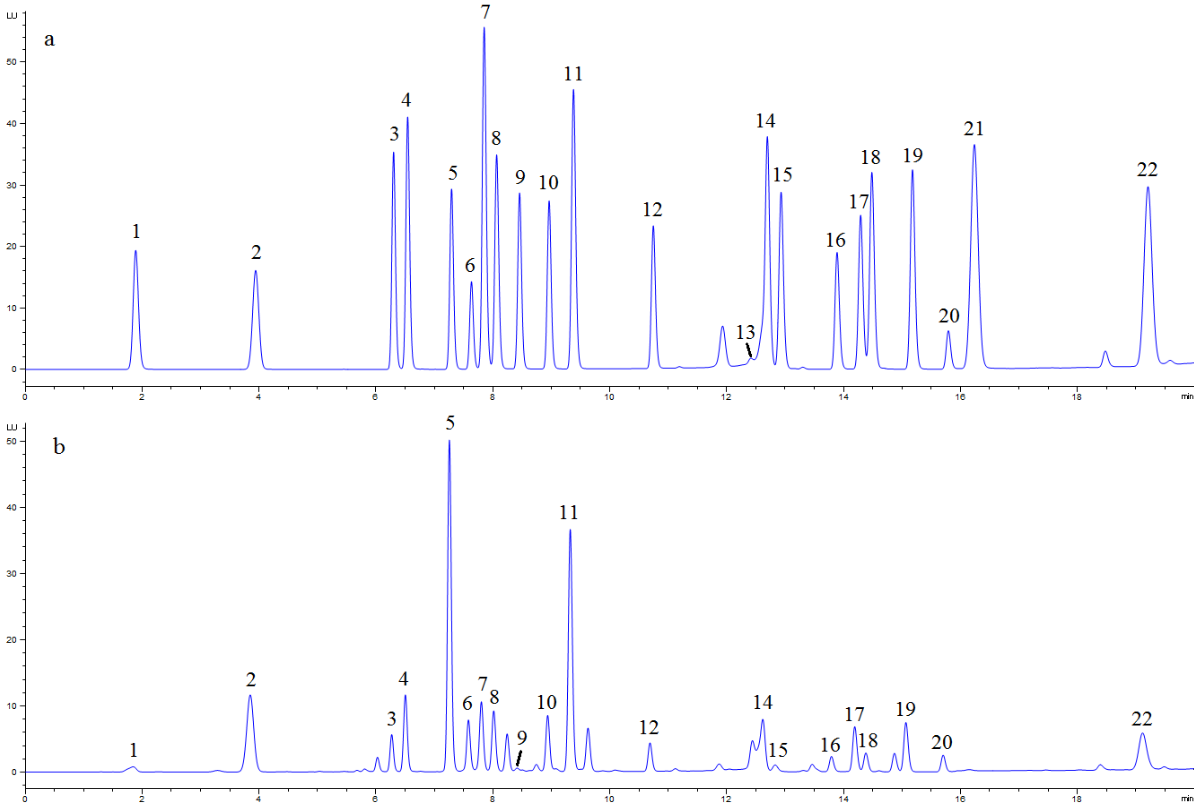

Figure S2. High performance liquid chromatography of amino acids analyzed by online OPA-FMOC derivation. a. Standard amino acids; b. Free amino acids of *L. deliciosus*. Peak identification: 1. Asparaginic acid; 2. Glutamic acid; 3. Asparagine; 4. Serine; 5. Glutamine; 6. Histidine; 7. Glycine; 8. Threonine; 9. Citrulline; 10. Arginine; 11. Alanine; 12. Tyrosine; 13. Cystine; 14. Valine; 15. Methionine; 16. Tryptophan; 17. Phenylalanine; 18. Isoleucine; 19. Leucine; 20. Lysine; 21. Hydroxyproline; 22. Proline.

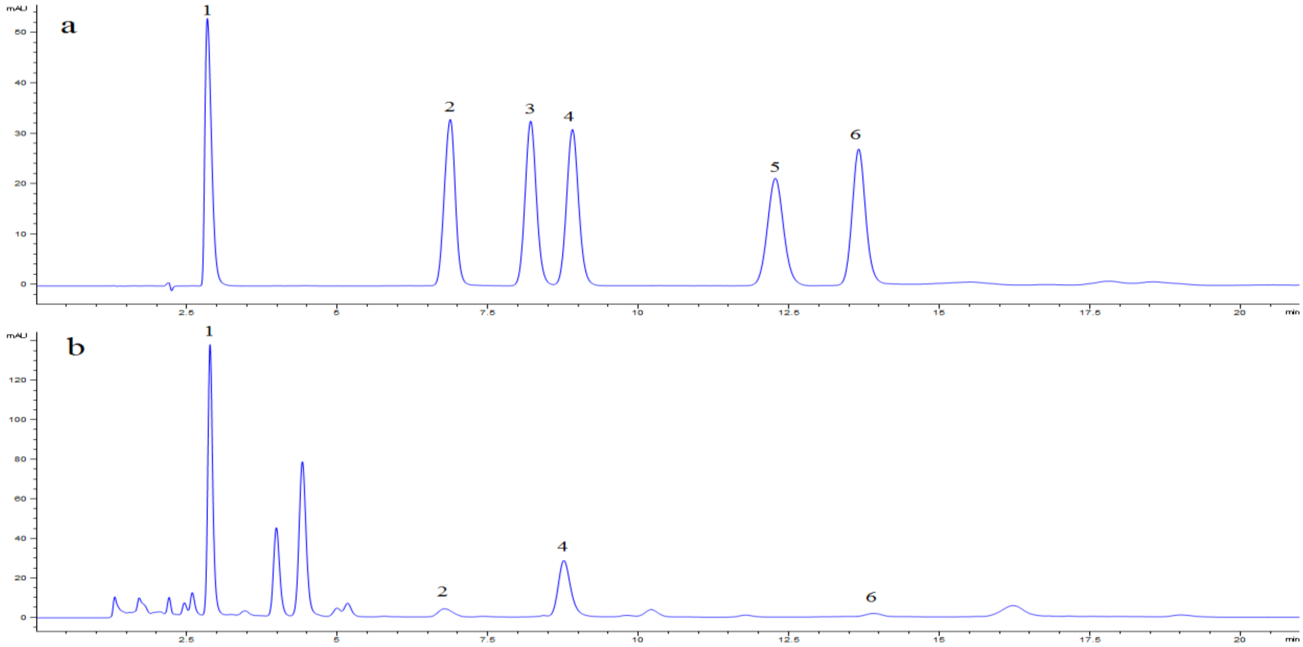

Figure S3. High performance liquid chromatography of flavor 5′-nucleotides. a. Standard 5′-nucleotides; b. 5′-nucleotides of *L. deliciosus*. Peak identification: 1. 5′-CMP; 2. 5′-UMP; 3. 5′-GMP; 4. 5′-IMP; 5. 5′-XMP; 6. 5′-AMP.

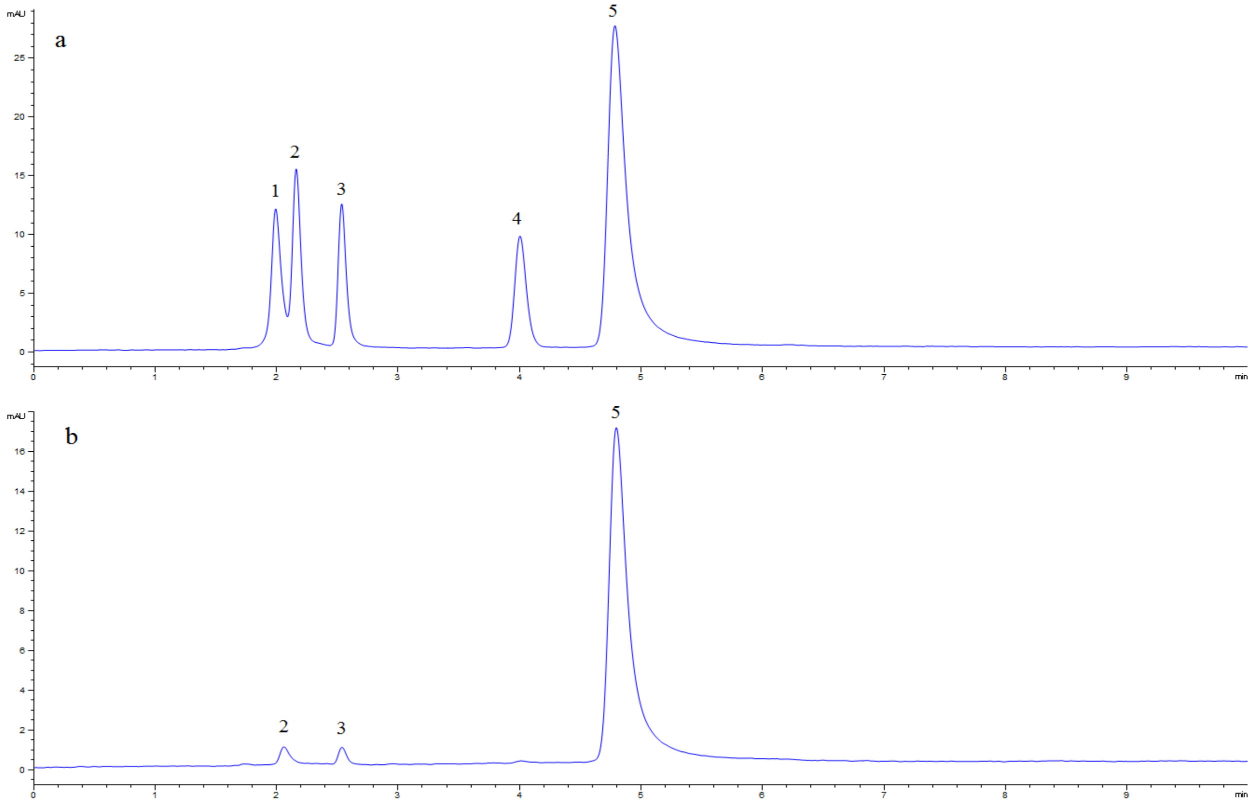

Figure S4. High performance liquid chromatography of organic acids. a. Standard organic acids s; b. organic acids of *L. deliciosus*. Peak identification: 1. Oxalic acid; 2. Quininic acid; 3. L-Malic acid; 4. Citric acid; 5. Fumaric acid.

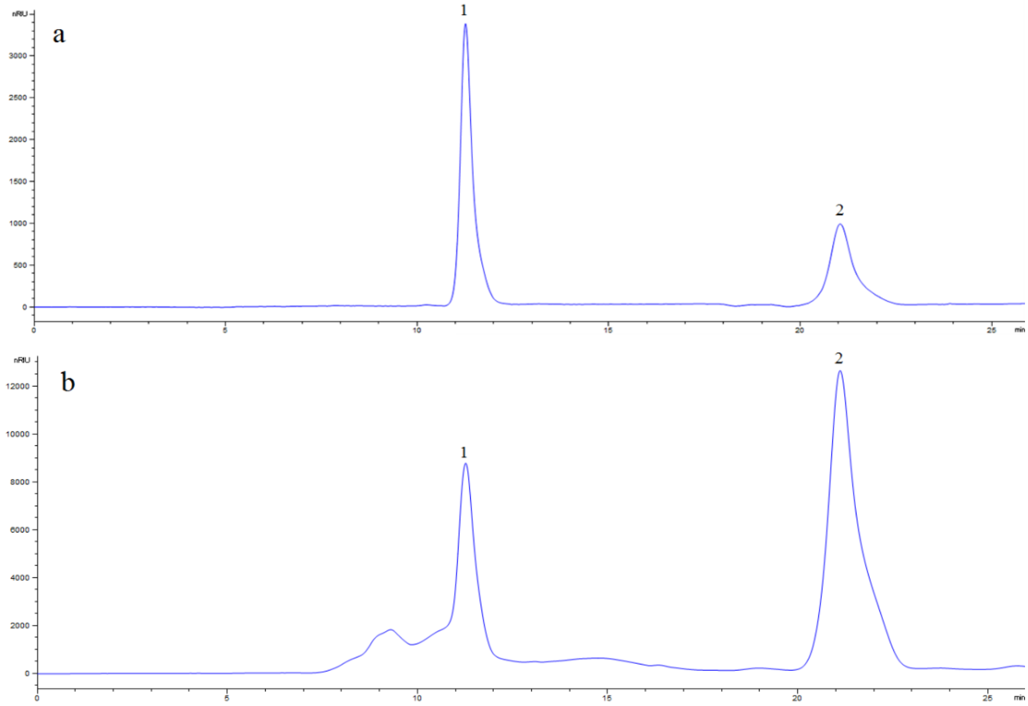

Figure S5. High performance liquid chromatography of free sugars. a. Standard free sugars s; b. free sugars of *L. deliciosus*. Peak identification: 1. Trehalose; 2. Mannitol.

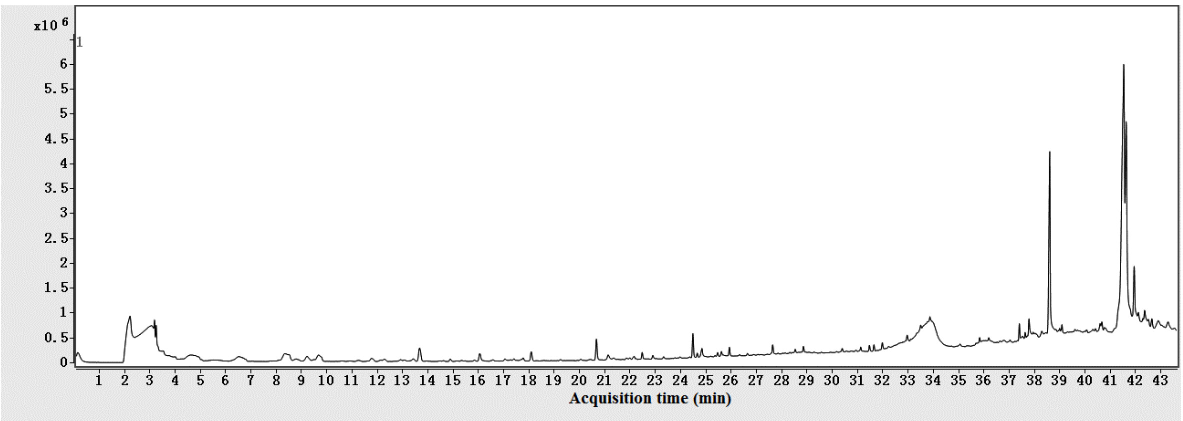

Figure S6. Headspace solid phase micro-extraction gas chromatography mass spectrum of volatile aroma compounds from *L. deliciosus*
